# Two-Photon-Induced Microstereolithography of Chitosan-*g*-Oligolactides as a Function of Their Stereochemical Composition

**DOI:** 10.3390/polym9070302

**Published:** 2017-07-24

**Authors:** Tatiana S. Demina, Kseniia N. Bardakova, Nikita V. Minaev, Eugenia A. Svidchenko, Alexander V. Istomin, Galina P. Goncharuk, Leonid V. Vladimirov, Andrey V. Grachev, Alexander N. Zelenetskii, Peter S. Timashev, Tatiana A. Akopova

**Affiliations:** 1 Enikolopov Institute of Synthetic Polymer Materials, Russian Academy of Sciences, 70 Profsoyuznaya str., Moscow 117393, Russia; evgensv@yandex.ru (E.A.S.); c6h5nh2@yandex.ru (A.V.I.); duna2011@yandex.ru (G.P.G.); anzel@ispm.ru (A.N.Z.); Akopova@ispm.ru (T.A.A.); 2 Institute of Photonic Technologies, Research center “Crystallography and Photonics”, Russian Academy of Sciences, 2 Pionerskaya str., Troitsk, Moscow 142190, Russia; arie5@yandex.ru (K.N.B.); minaevn@gmail.com (N.V.M.); timashev.peter@gmail.com (P.S.T.); 3 Institute for Regenerative Medicine, Sechenov University, 8-2 Trubetskaya st., Moscow 119991, Russia; 4 Semenov Institute of Chemical Physics, Russian Academy of Sciences, 4 Kosygina str., Moscow 119334, Russia; leo_v_jp@yahoo.com (L.V.V.); andrgrachyov@yandex.ru (A.V.G.)

**Keywords:** laser stereolithography, chitosan, lactide, mechanochemistry, two-photon polymerization, graft-copolymers, hydrogels

## Abstract

Chitosan-*g*-oligolactide copolymers with relatively long oligolactide grafted chains of various stereochemical compositions have been synthetized via a solvent-free mechanochemical technique and tailored to fabricate three-dimensional hydrogels using two-photon induced microstereolithography. An effect of the characteristics of chitosan and oligolactide used for the synthesis on the grafting yield and copolymer’s behavior were evaluated using fractional analysis, FTIR-spectroscopy, dynamic light scattering, and UV-spectrophotometry. The lowest copolymer yield was found for the system based on chitosan with higher molecular weight, while the samples consisting of low-molecular weight chitosan showed higher grafting degrees, which were comparable in both the cases of l,l- or l,d-oligolactide grafting. The copolymer processability in the course of two-photon stereolithography was evaluated as a function of the copolymer’s characteristics and stereolithography conditions. The structure and mechanical properties of the model film samples and fabricated 3D hydrogels were studied using optical and scanning electron microscopy, as well as by using tensile and nanoindenter devices. The application of copolymer with oligo(l,d-lactide) side chains led to higher processability during two-photon stereolithography in terms of the response to the laser beam, reproduction of the digital model, and the mechanical properties of the fabricated hydrogels.

## 1. Introduction

Hydrogels, i.e., cross-linked hydrophilic polymers that are able to retain a large amount of water, up to thousands of times of their own volume, are considered as promising scaffolds for the regeneration of various types of tissue. However, the hydrogels designed to work as scaffolds for tissue engineering should have an appropriate architecture. The macroporous structure of hydrogels allows for unobstructed transfer of nutrients, waste products, and cell migration, as well as an ability to serve as a guide for cell growth and, thus, to control tissue formation. In order for the tissue to be properly restored/substituted, a more complex structure of hydrogel should be required. There are a number of various methods to fabricate hydrogels with porous 3D architectures. However, the most often employed freeze-drying method, as well as some another techniques, e.g., gas foaming and supercritical fluids, usually result in only partial control over the hydrogel morphology. Fabrication of structures on micro- and nano-levels requires the application of techniques providing a generation of in vitro and/or in vivo tissue analogous structures.

Laser stereolithography allows the fabrication of 3D scaffolds with well-defined architectonics using CAD models [1,2]. Being a technique of additive manufacturing, laser stereolithography relies on layer-by-layer building of the materials by local solidification under laser-induced reactions. As a function of the features of the laser beam, resolution can reach submicron levels. For example, two-photon (2PP) stereolithography based on focused femtosecond laser pulses allows the creation of structures with a sub-diffraction limit of resolution, as well as overcoming local overheating, thus, providing an opportunity to immobilize sensitive components (proteins, cells, etc.) during the fabrication process [3]. However, the main challenge of this technique is the material, which is able to solidify under photo-induced reactions, as well as its suitability for biomedical applications. Recent efforts were made in the development of photopolymerizable resins based on natural polymers, such as hyaluronic acid, chitosan, gelatin, etc. [4,5]. Laser-assisted fabrication of structured hydrogels is also possible using 3D bioprinting [6,7,8].

Indeed, polysaccharides could be considered as ideal polymers to fabricate hydrogels for tissue engineering. For example, chitosan, a derivative of naturally-occurring chitin, is widely used for fabrication of scaffolds and drug delivery systems [9]. Chitosan possesses a range of prospective properties, such as biocompatibility, an ability to degrade under the action of enzymes presented in the human body, i.e., lysozyme, and versatility from the view point of hydrogel fabrication. Due to the presence of hydroxyl and amino groups in the chitosan structure, it can be cross-linked through covalent bonding by the use of various agents (glutaraldehyde, genipin, etc.) or physical bonding with small anionic molecules, metal anions, and negatively-charged polyelectrolytes [10,11]. On the other hand, these reactive groups could also act as sites for chemical modification and, therefore, to provide an ability to precisely control the final properties of chitosan-based hydrogels (mechanical stability, drug loading effectiveness, swelling, sensitivity to pH, temperature, etc.) [11]. Thus, modification of the polymer chemical structure could benefit in two ways: as a way to control the properties of chitosan-based materials, or broadening the number of methods of chitosan processing, i.e., hydrogel fabrication techniques.

The non-modified chitosan was used as a part of the photosensitive composition for laser stereolithography, but appeared to be a non-reactive “guest” [12]. The targeted chemical modification of chitosan for laser stereolithography was successfully carried out using polyvinyl alcohol, allyl bromide, and glycidyl methacrylate in [13,14,15,16]. This work was aimed to synthesize chitosan copolymers having the relatively long (degree of polymerization up to 70) grafted oligolactide chains of various stereochemical compositions and to evaluate their effectiveness for 2PP-induced microstereolithography. Since polylactides have an extraordinary versatility in terms of material properties, their applicability in processing technologies, as well as a successful, well-established history in biomedical applications, their application for modification of the chitosan chemical structure could provide more benefits than methacrylated systems [17,18,19]. Previously, the grafting of short oligo(l,d-lactide) chains onto chitosan was shown to be an effective approach to control hydrogel properties (biocompatibility, biodegradation rate, mechanical properties) and to provide the reaction ability for laser-induced reactions [20,21]. An increase in the degree of polymerization (from three to 10) of the grafted oligolactide chains led to an increase in the effectiveness of 2PP-stereolitography [22]. Here, we synthetized graft-copolymers of chitosan with relatively long oligolactide side chains (with a degree of polymerization up to 70) of various stereochemical composition and evaluated the effect of copolymer characteristics on the processability for 2PP-induced microstereolithography and mechanical characteristics of the fabricated hydrogels. This combination of chitosan and oligolactide could allow the fabrication of scaffolds for tissue engineering with well-defined architecture, enhanced biocompatibility, and controllable mechanical and swelling properties, as well as having a biodegradation rate as a function of the copolymer’s characteristics and processing conditions.

## 2. Experimental

### 2.1. Materials and Copolymers Processing

**Figure 1:**
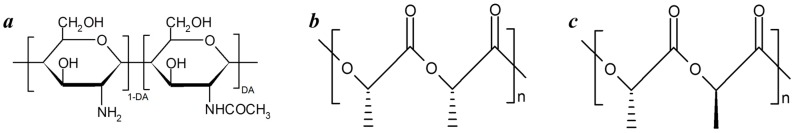
Chemical formulas of the initial chitosan (**a**); oligo(l,l-lactide) (**b**); and oligo(l,d-lactide) (**c**).

Chitosan-*g*-oligo(l,l-/l,d-lactide) copolymers were prepared by mechanochemical treatment of solid powder mixtures of chitosan and oligo(l,l-lactide) or oligo(l,d-lactide) in a Berstorff ZE-40 semi-industrial twin-screw extruder (KraussMaffei Berstorff, Munich, Germany) at 55 °C. Chemical structures of chitosan and oligo(l,l-/l,d-lactides) are shown in Figure 1. For the synthesis, two chitosan samples were used. Chitosan (marked as Chs-c) with an average molecular weight M_w_ of 350 kDa and degree of acetylation DA of 0.14 was purchased from Sonat (Russia). Chitosan (marked as Chs-s) with M_w_ of 80 kDa and DA of 0.11 was prepared from crab chitin supplied by Xiamen Fine Chemical (Xiamen, China) through the solid-state mechanochemical synthesis in ISPM RAS (Moscow, Russia) as reported earlier [23]. Semi-crystalline oligo(l,l-lactide) and amorphous oligo(l,d-lactide) with M_w_ of 5000 were synthesized from respective lactic acids (Panreac, Spain) using 0.001% SnCl_2_ as a catalyst. Conditions of the solid-state synthesis of the chitosan-*g*-oligo(l,l-/l,d-lactide) copolymers are listed in Table 1 (see Section 3.1).

Poly(ethylene glycol) diacrylate (PEG-DA, Sigma-Aldrich, M_w_ of 2000) and Irgacure 2959 photoinitiator (BASF Kaisten AG, 98% purity) were used as additional components of the photosensitive compositions.

### 2.2. Characterization of Chitosan-g-oligo(l,l-/l,d-lactide) Copolymers

The percentage of oligolactide linked to chitosan was calculated from the difference in weight observed between copolymer samples after their purification by acetone and oligolactide initially taken for synthesis. Purification of the samples was carried out as follows: a sample (about 0.7 g) was dispersed in 25 mL of acetone for 2 h at room temperature (RT) under magnetic stirring. After dissolution of unreacted oligolactide, the insoluble fraction was collected by filtration, washed several times on paper filters with acetone, and dried in a vacuum oven (Labtex, Moscow, Russia). The grafting percentage was calculated as:

(1) $$\frac{W_{\mathrm{cl}}-W_{\mathrm{chs}}}{W_{\mathrm{chs}}}\times 100,$$

where $W_{\mathrm{cl}}$ is theweight of copolymer after purification from unreacted oligolactide, and $W_{\mathrm{chs}}$ is the weight of chitosan initially taken for the synthesis.

The ability of the copolymers to dissolve in aqueous media was evaluated in deionized water and 2% CH_3_COOH as follows: a sample (about 0.7 g) was dissolved by stirring in 70 mL of water or 2% acetic acid at RT for 2 h. The insoluble fractions were separated by centrifugation, repeatedly washed with deionized water, freeze-dried, and weighted. Water soluble fractions were precipitated with 1 M NaOH, collected by centrifugation, washed with deionized water and freeze-dried.

Infrared spectra were recorded on a Bruker Vertex 70 spectrometer (USA). All spectra were initially obtained in Attenuated Total Reflectance (ATR) mode at a resolution of 2 or 4 cm^−1^ by using an ATR-mono-reflection GladiATR (Pike Technologies, Madison, WI, USA) accessory equipped with a monolithic diamond single-reflection crystal (angle of incidence −45°, refractive index = 2.4). The thus-obtained ATR spectra were further converted into IR-absorbance modes. All the spectra presented in this work were recorded and treated using the program Bruker Opus (version 6.0, Bruker, Billerica, MA, USA) The spectra were normalized with respect to the composite stretching band at 1080 cm^−1^—the strongest band of the envelope of overlaid C–O bands [C–O–C and C–O(H)].

The hydrodynamic diameters of the chitosan and copolymer aggregates generated in 2% acetic acid were determined by dynamic light scattering (DLS) using a Zetatrac particle size analyzer (Microtrac, Inc., Montgomeryville, PA, USA) with the Microtrac application software program (version 10.5.3, Microtrac, Inc., Montgomeryville, PA, USA). The polymer solutions (0.1 wt %) were prepared under magnetic stirrer agitation for 2 h at RT.

UV spectrophotometry of 1% solutions of non-modified chitosan and chitosan-*g*-oligo(l,l-/l,d-lactide) copolymers in 0.1 M HCl, as well as oligolactides in dichloromethane, was carried out in a quartz cells with an optical path length of 1 cm using a Shimadzu UV 2501 PC spectrophotometer. The analysis of spectral data was carried out after subtracting the contribution of corresponding solvent and mathematical separation of the bands related to absorption and Rayleigh scattering.

### 2.3. Fabrication of the Hydrogels by Two Photon-Induced Microstereolithography

Non-modified chitosans and the synthesized copolymers were used as a base for photosensitive compositions and evaluated for their processability using 2PP-induced microstereolithography. For that, the copolymers were dissolved in 2% CH_3_COOH to achieve 4–4.5 wt % concentration and mixed with PEG-DA and photoinitiator. The final photosensitive compositions contained 4–4.5 wt % of chitosan copolymer, 6.4–7.1 wt % of PEG-DA and 1 wt % of photoinitiator. The prepared photosensitive compositions were transferred to a silicon spacer, cover-slipped, and underwent structuralization under a laser beam.

**Figure 2:**
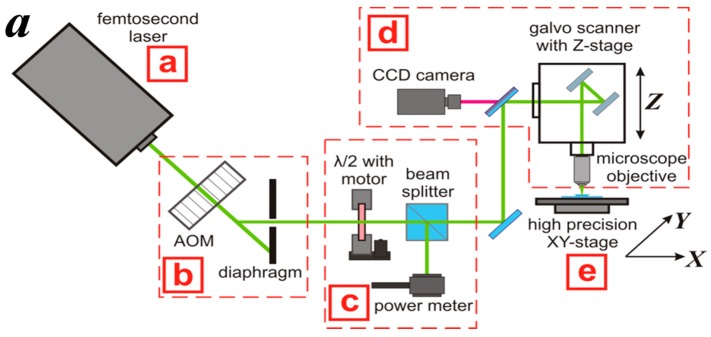

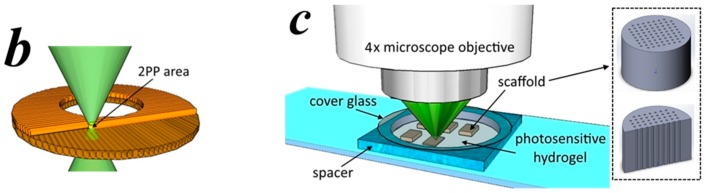
Schematic diagram of the 2PP microstereolithography setup (see description in the text) (**a**) principle of layer-by-layer scaffold structuralization; (**b**) the experimental system for hydrogel fabrication; and the 3D scaffold model (**c**).

We used a ytterbium-doped femtosecond solid-state laser “TeMa-100” with a second harmonic generator (Avesta-Project, Troitsk-Moscow, Russia) as a source of femtosecond laser pulses with a wavelength of 525 nm, a pulse duration of green femtoimpulses was about 200 fs, and a pulse repetition rate of 70 MHz (Figure 2(aa)). An optic gate (Figure 2(ab)) acts as an acousto-optic modulator, allowing the laser beam to turn on and off laser with a frequency greater than 1 MHz. To control the power of laser radiation (Figure 2(ac)) we use a half-wave plate positioned on a motorized rotatable stage and a polarizing beam splitter cube. A power meter mounted on the side of the beam splitter was used for the continuous monitoring of laser power arriving in the photoresist volume.

To transfer the laser beam to the targeted photosensitive composition spot the system was mounted on a precision Z-stage translator, consisting of a galvo scanner with a 4× PLAN objective (Figure 2(ad)). In contrast to the 20× objective previously used in [22], this objective allows to us to fabricate larger 3D structures with well-defined architectonics, which are more relevant for biomedical applications. The galvo scanner allowed the high-speed transfer of the focused laser beam in the plane of the objective’s field of view (diameter: 1000 μm). A charge coupled device (CCD) camera provided an ability to focus the laser beam, as well as to observe the structuralization process. The sample was placed on the precision XY-stage translator (Figure 2(ae)) allowing movement with submicron resolution. The laser beam was focused in a voxel having a shape of an ellipsoid with a Z-height of 15 μm and an X-Y diameter of 6 μm.

During the 2PP-stereolithography each horizontal layer was formed perpendicularly to the previous one and overlapped it (Figure 2b). The value of the overlapping was varied using the parameter *Z-Slice*, i.e., the vertical distance between voxel centers. Each layer consisted of parallel lines, which were formed using various parameters of XY-hatch, i.e., the horizontal distance between voxel centers. The effect of the variation of Z-Slice and XY-hatch was evaluated during the fabrication of 3D hydrogels in a form of cylinders with a diameter of 1 mm, a height of 0.5 mm, and pore diameter of 50 μm (Figure 2c). The image files of the 3D model used for the laser stereolithography can be found in the supplementary file (Figure S1). The fabricated 3D hydrogels were washed from the uncured material in deionized water for 4–5 h.

### 2.4. Characterization of the Hydrogels

The mechanical properties of the fabricated hydrogels, as well as model film samples, were evaluated. Model films were cast from 2 wt % polymer solutions in 2% acetic acid on polystyrene Petri dishes, and then dried in a dust-free chamber at RT (about 48 h). Mechanical properties of the film samples were evaluated using an AGS-H universal tensile machine (Shimadzu, Kyoto, Japan) at a speed of 1 mm/min.

The bulk and surface morphology of the model films and the hydrogels was studied using optical microscopy (HRM-300 (Huvitz, Gunpo, Korea)) and scanning electron microscopy (PhenomProX (PhenomWorld, Eindhoven, The Netherlands)).

Mechanical properties of the hydrogels were studied using a Piuma NanoIndenter (Optics11, Amsterdam, The Netherlands) [24]. The Young’s modulus of the hydrogels surface was evaluated using a cantilever with a hardness of 0.46 H/m and tip radius of 27.5 μm at 22 °C. The measurements were carried out on five various areas (100 × 100 μm) of each scaffold at a resolution of 20 μm.

## 3. Results and Discussion

### 3.1. Copolymer Characterization

Since the synthesized products consist of hydrophilic and hydrophobic chains, the obtained systems possessed amphiphilic properties and had an affinity to both aqueous and chlorinated solvents. Dissolution of the samples in classical oligo/polylactide solvents, such as chloroform or dichloromethane, led to their swelling and formation of ultra-fine stable dispersions. Therefore, the purification of samples from unreacted oligolactides was carried out using acetone, which serves as a good solvent for oligo/polylactides and as precipitating agent for chitosan. The calculation of amounts of the reacted oligolactide and the corresponding grafting degrees are shown in the Table 1. The lowest reactivity was found for the system based on Chs-c (CLL-c), while the samples consisting of chitosan with lower M_w_ (Chs-s) showed higher grafting degrees, which were comparable in the case of l,l- or l,d-oligolactides. The difference in the reactivity as a function of chitosan M_w_ could be caused by the accessibility of the chitosan function groups during the solid-state mechanochemical treatment.

**Figure 3:**
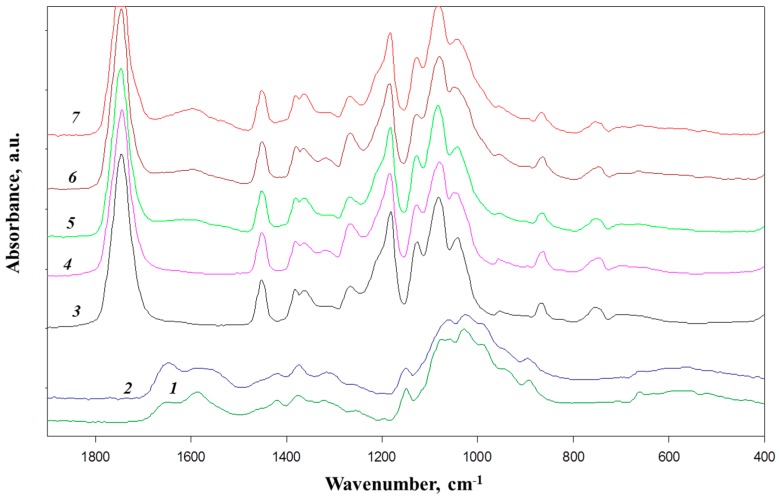
FTIR spectra of Chs-s (1), Chs-c (2), oligo(l,l-lactide) (3), and oligo(l,d-lactide) (4), CLL-c (5), CLD-s (6), and CLL-s (7) samples.

The FTIR spectra of the synthesized products as well as the spectra of the initial components are shown in Figure 3. The DA of commercial chitosan sample (marked as Chs-c) is substantially higher than that of Chs-s, which can be seen from comparison of intensities of amide I bands (1653 cm^−1^) with the bands of the bending vibrations of the NH_2_ groups (1590 cm^−1^) in the spectra of the initial chitosan samples [25]. This is in good agreement with DA data obtained by using potentiometric titration and ^1^H NMR.

The FTIR spectra of the oligolactides and the copolymers contain a full set of bands characteristic of lactide chains, most typical of which are: 1747 cm^−1^—the stretching of C=O of ester group; a doublet of bands 1380 and 1363 cm^−1^—with a high contribution of symmetric deformation modes of CH_3_ groups; 1183 cm^−1^—a relatively strong band of asymmetric C–O–C stretching; 1083 cm^−1^—symmetric CH_3_ stretching and 1063 cm^−1^ of C–C stretching [26]. The band at 1452 cm^−1^ could be attributed to asymmetric deformation of CH_3_ groups and it is almost insensitive to the physical state of the lactic chain and, thus, can be used as an internal standard for evaluation of the degree of crystallinity [27]. The studies of crystallization behavior of poly(l-lactic acid) revealed a group of bands possessing the highest sensitivity to the extent of crystallinity of PLLA: 1363 and 1210 cm^−1^ bands [27]. The latter corresponds to a combination of asymmetric C–O–C and asymmetric rocking vibrations of CH_2_ group and in samples with a low degree of crystallinity can be seen as a shoulder of the band at 1083 cm^−1^. Based on the above band assignments it is clearly seen from the spectra presented in Figure 3 that the oligo(l,d-lactide) (Figure 3, spectrum 4) is fully amorphous, while oligo(l,l-lactide) (Figure 3, spectrum 3) possesses a rather low degree of crystallinity. The crystalline features of initial oligolactides remain unchanged after the grafting procedure: the oligolactide grafts of CLL-c sample (Figure 3, spectrum 5) have a higher crystallinity, whereas oligolactide chains of CLD-s (Figure 3, spectrum 6) are fully amorphous. It also follows from Figure 3 that the CLL-s sample (Figure 3, spectrum 7) shows the highest relative intensity of the “crystallinity-bands”.

The FTIR spectra of the copolymers also have several distinctions as compared with the spectra corresponding to initial oligolactides. These differences are most clearly manifested in the spectrum of CLL-s (Figure 3, spectrum 7), in particular, in the appearance of a low-frequency shoulder at approx. 1710 cm^−1^ attributed to the bands of carboxyl groups and a weak broad band with a maximum at approx. 1600 cm^−1^. In this frequency range lie the bands of asymmetric stretching vibrations COO–groups, as well as of deformation vibrations of NH_3_^+^ groups. The large (about 80 cm^−1^) half-width of this band suggests that the mentioned bands are superimposed. Thus, it is logical to assume that the band at 1600 cm^−1^ reflects the formation of a salt from COO–NH_3_^+^ bonds. The low intensity of the band at 1600 cm^−1^ indicates that the changes of properties of the oligolactide-grafted chitosans require a very low concentration of salt cross-links. The intensity of this band in the spectra of CLL-c and CLD-s (Figure 3, spectra 5 and 6) is lower than that for the CLL-s sample, which corresponds with lower amount of grafted oligolactide chains (see Table 1).

Since the copolymers were intended to be used for hydrogel fabrication, their solubility in aqueous media was evaluated as well. Grafting of hydrophobic oligolactide chains led to an anticipated decrease in the copolymers’ solubility in aqueous solutions: CLL-s and CLD-s consisted of 49 and 58 wt % of insoluble 2% acetic acid fractions, respectively. The low grafting degree of the CLL-c sample led to a better solubility in aqueous acetic acid: the amount of insoluble fraction was 40 wt %.

**Figure 4:**
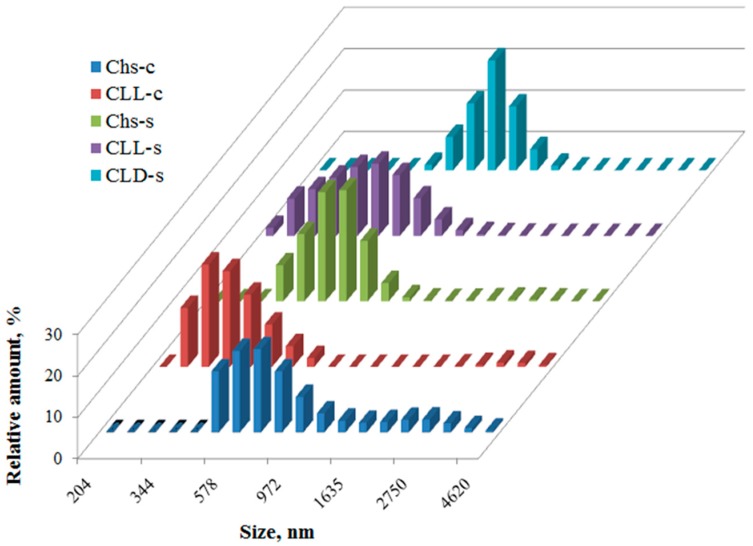
Number-weighted size distribution in 0.1 wt % solutions of non-modified chitosan and the copolymers in 2% CH_3_COOH.

According to the DLS data, the mean size of chitosan associates increased when the molecular weight of chitosan was smaller. The presence of oligo(l,l-lactide) fragments led to decrease in associates size (CLL-c and CLL-s), while the copolymer with oligo(l,d-lactide) has hydrodynamic diameters greater than non-modified chitosan Chs-s (Figure 4). This difference may be caused by various contributions of intra-/intermolecular interactions of polymeric chains in the solutions. The l,l-fragments promote the intramolecular interactions between grafted oligo(l,l-lactide) side chains, while grafting of oligo(l,d-lactide) stimulates the intermolecular forces between copolymer macromolecules.

**Figure 5:**
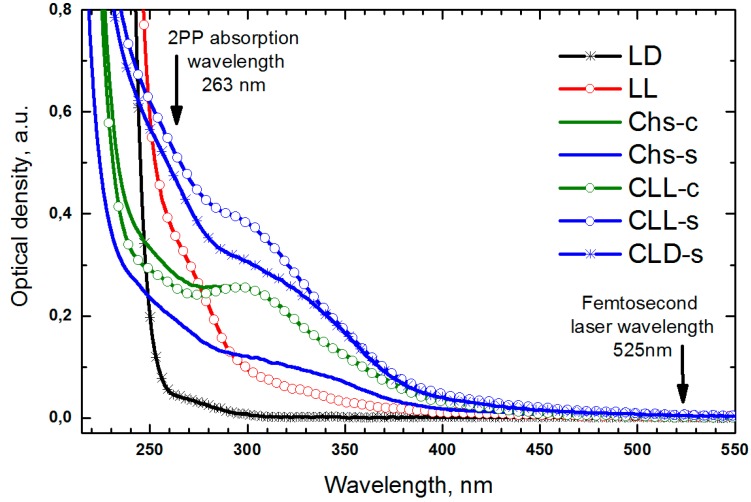
Electron absorption spectra of 1 wt % solutions of oligo(l,l-lactide) and oligo(l,d-lactide) in CH_2_Cl_2_; non-modified Chs-s, Chs-c; CLL-c, CLL-s, and CLD-s copolymers in 0.1 M HCl.

The UV-spectrophotometry data were in a good agreement with the results of calculation of the grafting degree and the DLS analysis. As it could be seen from Figure 5, the grafting of oligo(l,l-lactide) to Chs-c backbone did not lead to any significant changes in the electronic spectra, which is in good accord with the low grafting degree of the CLL-c sample. Whereas the grafting of both oligolactides to Chs-s chains led to an increase in the intensity of bands in a range of 200–400 nm (cf. the curves for CLL-s, CLD-s, and Chs-s samples in Figure 5). It was shown earlier in [12] that these changes should be attributed to the reacted chitosan amino groups. The bands’ intensities of the CLL-s and CLD-s spectra, as well as their shapes are close to the superposition of the spectra of initial components. As compared with non-modified chitosan, the short wavelength band (<250 nm) appears in the spectra of copolymers as a result of the significant amount of oligolactide incorporated in the Chs-s structure (see Table 1). The band at 320 nm, which was found in oligo(l,l-lactide)-based samples, could be attributed to interaction between semi-crystalline fragments, which is in agreement with DLS data.

As a whole, electronic absorption spectra of the samples showed no absorbance at the laser wavelength (525 nm) providing a possibility to precisely focus a laser beam and to fabricate copolymer-based hydrogels by 2PP-laser stereolithography.

### 3.2. Two-Photon Induced Stereolithography of the Copolymers

The effect of the copolymers’ characteristics, as well as the stereolithography conditions on the efficiency of the 2PP-induced cross-linking process and properties of the fabricated hydrogels, was evaluated. The photosensitive compositions based on non-modified chitosans and synthesized copolymers were used for 2PP-stereolithography at various processing conditions. The effectiveness of 2PP was evaluated using three main criteria, i.e., (1) the response to the laser beam (cross-linking); (2) reproducing the digital model; and (3) sufficient mechanical stability (material integrity) after washing off the unreacted fragments from the final structures.

The variation of the processing conditions such as XY-hatch and Z-slice, i.e., the distances between the lines and layers, allows us to control stereolithography productivity and the properties of the fabricated hydrogels. For example, an increase of XY-hatch and Z-Slice from 5 to 7 μm led to a significant increase of required time: 5 min for XY-hatch = 5, Z-slice = 5, and 1.5 min for XY-hatch = 7, Z-slice = 7. However, the significant distances between the lines and layers could lead to a lack of elemental unit connection and, thus, to an inability to create an integral 3D structure. The optimization of XY-hatch and Z-slice allows us also to control the microtopography of hydrogels and their cross-linking density and, thus, their swelling parameters.

**Figure 6:**
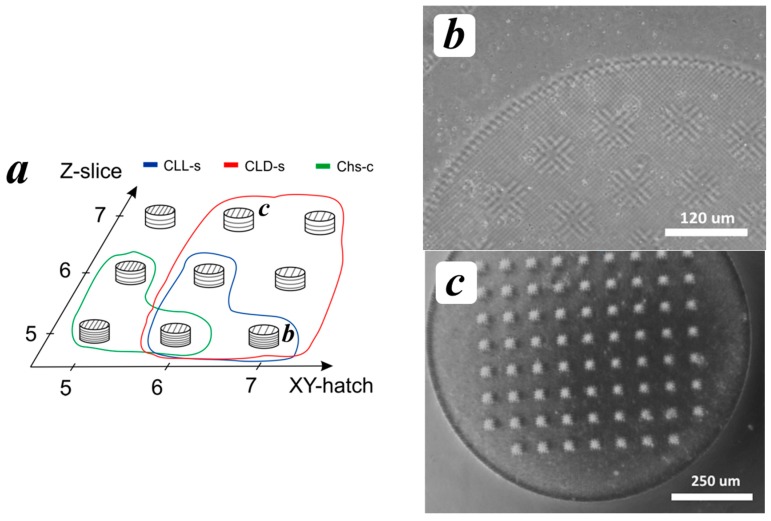
Optimal parameters of the 2PP process for various polymers (**a**); optical micrograph of the scaffold based on CLL_s, *Z-Slice* = 5, *XY-hatch* = 7 (**b**); and the scaffold based on CLD_s, *Z-Slice* = 7, *XY-hatch* = 6 (**c**).

The screening of the polymeric systems showed that only the photosensitive compositions based on Chs-c, CLL-s, and CLD-s were suitable for the 2PP cross-linking process. Optimal values of XY-hatch and Z-slice for each viable polymeric system are shown in Figure 6a. The usage of the Chs-c composition allowed obtaining mechanically-stable hydrogels only at lower values of XY-hatch/Z-slice, i.e., at the highest laser beam density. The Chs-c gave a rise in the reproducible structures, which increased in volume up to 3.5–4 times during washing. In contrast to Chs-c, the CLL-c sample (copolymer of Chs-c with oligo(l,l-lactide)) also possessed sufficient reactivity, but the structures were completely destroyed during the washing even after processing at XY-hatch/Z-slice = 3 μm.

The photosensitive compositions based on chitosan Chs-s with lower M_w_ and DA could not provide sufficient reactivity under the laser beam to obtain even a stable elemental unit (line). The usage of the copolymer with oligo(l,l-lactide) (CLL-s) allowed the fabrication of structures under the laser beam, but the obtained hydrogels showed rather low reproducibility of the digital model and poor mechanical properties. The most successful photosensitive composition was based on the copolymer of Chs-s with oligo(l,d-lactide) (CLD-s) allowing the creation of well-defined 3D-stable hydrogels at XY-hatch = 6–7 μm in the whole range of Z-slice. The prepared hydrogels were mechanically stable during the washing and showed a two-fold volume increase. In fact, the CLD-s is an optimized version of chitosan-*g*-oligolactide copolymer discussed in [22], where the polymerization degree of the grafted oligo(l,d-lactide) fragments varied in a range of 3–10. Here, the increase of oligolactide chain length allowed us to decrease the amount of PEG-DA. The variation of length and stereochemical composition of the grafted chains could also be of benefit in terms of hydrogel characteristics, such as mechanical properties, biocompatibility, and biodegradation rate.

The mechanism of the cross-linking of chitosan and its copolymers with oligo/polylactides during 2PP-induced microstereolithography could proceed through various reaction channels. We assume that PEG-DA works mainly as flexible spacers between chitosan chains, since its concentration in the photosensitive composition is too low to achieve a monolithic structure. Reaction channels should work equally for the photosensitive compositions containing either l,l- or l,d-oligolactide fragments. However, the processability of CLL-s and CLL-c-copolymers was significantly limited in comparison with CLD-s, which could be caused by different self-assemblies of the copolymers containing either l,l- or l,d- oligolactide side chains .

**Figure 7:**
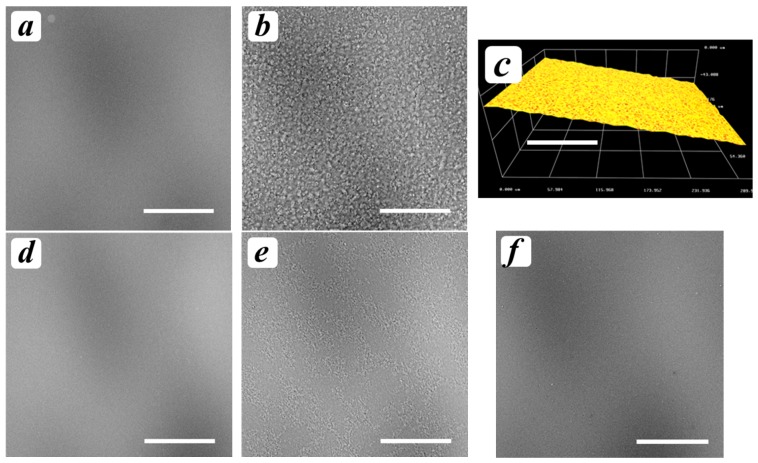
SEM (**a**,**b**,**d**–**f**) and microscopy (**c**) images of the model films cast from Chs-c (**a**), CLL-c (**b**,**c**), Chs-s (**d**), CLL-s (**e**), and CLD-s (**f**). The scale bar is 100 μm.

The study of the model copolymers’ film structures using SEM and 3D microscopy showed that the film samples cast from non-modified chitosans (Figure 7a,d), as well as one of CLD-s (Figure 7f), possessed homogenous surface morphology, while the samples containing oligo(l,l-lactide) fragments (Figure 7b,e) had heterogeneous ones. As could be seen in Figure 7b,c, the surface and bulk morphology of the CLL-c sample showed a structure which could be attributed to a formation of chitosan film filled with oligolactide domains. Indeed, the acetic acid dissolves only chitosan fragments, which could stabilize the domains of hydrophobic oligolactides within the aqueous media. As it was previously shown using DLS and UV-spectrophotometry, the oligo(l,l-lactide) fragments promoted the interactions between grafted side chains and, thus, have the tendency to form oligolactide domains. Thus, the most heterogeneous CLL-c-based hydrogels were completely destroyed during washing in spite of sufficient reactivity under the laser beam.

**Figure 8:**
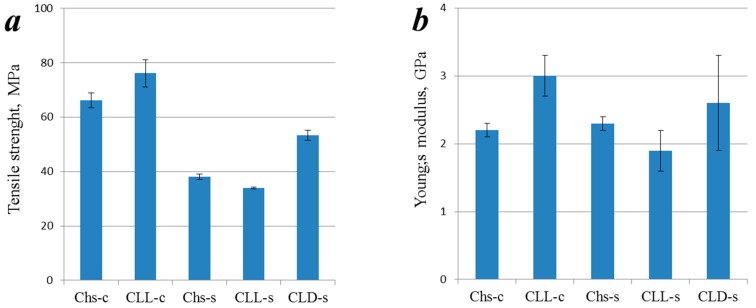
Tensile strength (**a**) and Young’s modulus (**b**) of the model films made of non-modified chitosan and its copolymers.

As can be seen from Figure 8, the presence of semi-crystalline oligo(l,l-lactide) fragments increased the strength of the model films in the case of grafting onto Chs-c, while grafting onto Chs-s led to а minor decrease of the film’s tensile strength. However, the grafting of oligo(l,d-lactide) side chains significantly (by 40%) increased the tensile strength. The film’s Young’s modulus has a similar tendency depending on the polymer’s characteristics.

**Figure 9:**
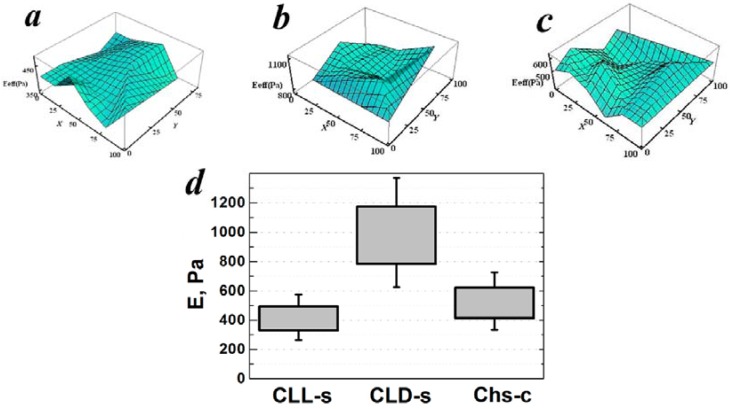
Distribution of Young’s modulus over surface area (100 μm × 100 μm) of hydrogels made of CLL-s (**a**), CLD-s (**b**), and Chs-c (**c**); mean values of the hydrogels’ Young’s modulus calculated by averaging the results obtained over five surface areas for each sample (**d**).

The values of the Young’s modulus of the hydrogels fabricated using laser stereolithography were in good agreement with the data of mechanical properties on the model films. The absolute values of the Young’s modulus for hydrogel samples were predictably lower than those for solid films, and showed the same tendency as a function of polymer characteristics. As can be seen in Figure 9 the highest Young’s modulus was found for the CLD-s-based sample (978 ± 176 Pa), which could be caused by properties of the polymer itself, or by the higher cross-linking density due to the better response to the laser beam action. The mean values of the Young’s modulus of CLL-s and Chs-c-based hydrogels were lower (411 ± 74 Pa and 518 ± 93 Pa, respectively) and its distributions over the hydrogel’s surfaces were narrower (Figure 9a,c).

Thus, as a function of the characteristics of the initial components, the grafting yields and processability of the synthetized graft-copolymers significantly varied. Optimized conditions of the copolymers’ syntheses and two-photon stereolithography allowed the fabrication of well-defined 3D structures with controlled mechanical properties. Since the chosen copolymers consisted of biocompatible and biodegradable components, they could be successfully employed for fabrication of 3D scaffolds for tissue engineering.

## 4. Conclusions

The graft-copolymers of biocompatible and biodegradable chitosan and oligolactide were synthetized via a solid-state mechanochemical technique. To control the effectiveness of the copolymerization, as well as properties and processability of the products, the characteristics of the chitosan backbone and oligolactide stereochemical composition were optimized. Dynamic light scattering and UV-spectroscopy were in good agreement with the calculation of the grafting degree and showed that the copolymers were suitable for application as polymer bases of photosensitive compositions for laser stereolithography. The copolymers’ processability under the action of laser radiation with femtosecond pulse duration was evaluated at various processing conditions. It has been found that the application of the copolymer made of chitosan with lower molecular weight and grafted amorphous oligo(l,d-lactide) chains allowed the fabrication of hydrogels with highly reproducible structures and optimized mechanical characteristics using two-photon stereolithography. The fabricated 3D hydrogels with well-defined architecture and enhanced biocompatibility could serve as scaffolds for tissue engineering.

## Figures and Tables

**Table 1:** List of the chitosan-*g*-oligolactide batches: conditions of the treatment and the copolymer’s yield.

Sample	Components	Components Ratio, *w*/*w*	Relative Amount of the Reacted Oligolactide, wt % *	Grafting Degree, %
CLL-c	Chs-c/oligo(l,l-lactide)	50/50	5.4	5.4
CLL-s	Chs-s/oligo(l,l-lactide)	40/60	23.4	35.1
CLD-s	Chs-s/oligo(l,d-lactide)	40/60	24.5	36.7

* Percentage of the reacted lactide amount was estimated as a ratio to initial lactide quantity taken for the synthesis. The grafting degree (%) was calculated as follows: ((graft-copolymer weight—chitosan weight)/chitosan weight) × 100.

## Supplementary Materials

The following are available online at http://www.mdpi.com/2073-4360/9/7/302/s1, Figure S1: 3D model used for the laser stereolithography.
