# Supplementary material for: Two-Photon-Induced Microstereolithography of Chitosan-g-Oligolactides as a Function of Their Stereochemical Composition
Source: Polymers (Basel). 2017 Jul 24;9(7):302. doi: 10.3390/polym9070302 (PMC6432183; doi:10.3390/polym9070302)
Supplement: Supplementary file 1 [file polymers-09-00302-s001.zip › cylinder_D1000_H500_d50 - 3D image.PDF]

Enable 3D View
